# Anti-Cancer Effect of HIV-1 Viral Protein R on Doxorubicin Resistant Neuroblastoma

**DOI:** 10.1371/journal.pone.0011466

**Published:** 2010-07-07

**Authors:** Richard Y. Zhao, Dong Liang, Ge Li, Christopher W. Larrimore, Bernard L. Mirkin

**Affiliations:** 1 Department of Pathology, University of Maryland School of Medicine, Baltimore, Maryland, United States of America; 2 Department of Microbiology-Immunology, University of Maryland School of Medicine, Baltimore, Maryland, United States of America; 3 Institute of Human Virology, University of Maryland School of Medicine, Baltimore, Maryland, United States of America; 4 Department of Pediatrics, Children's Memorial Institute for Education and Research, Northwestern University Feinberg School of Medicine, Chicago, Illinois, United States of America; George Mason University, United States of America

## Abstract

Several unique biological features of HIV-1 Vpr make it a potentially powerful agent for anti-cancer therapy. First, Vpr inhibits cell proliferation by induction of cell cycle G2 arrest. Second, it induces apoptosis through multiple mechanisms, which could be significant as it may be able to overcome apoptotic resistance exhibited by many cancerous cells, and, finally, Vpr selectively kills fast growing cells in a p53-independent manner. To demonstrate the potential utility of Vpr as an anti-cancer agent, we carried out proof-of-concept studies *in vitro* and *in vivo*. Results of our preliminary studies demonstrated that Vpr induces cell cycle G2 arrest and apoptosis in a variety of cancer types. Moreover, the same Vpr effects could also be detected in some cancer cells that are resistant to anti-cancer drugs such as doxorubicin (DOX). To further illustrate the potential value of Vpr in tumor growth inhibition, we adopted a DOX-resistant neuroblastoma model by injecting SK-N-SH cells into C57BL/6N and C57BL/6J-scid/scid mice. We hypothesized that Vpr is able to block cell proliferation and induce apoptosis regardless of the drug resistance status of the tumors. Indeed, production of Vpr *via* adenoviral delivery to neuroblastoma cells caused G2 arrest and apoptosis in both drug naïve and DOX-resistant cells. In addition, pre-infection or intratumoral injection of *vpr*-expressing adenoviral particles into neuroblastoma tumors in SCID mice markedly inhibited tumor growth. Therefore, Vpr could possibly be used as a supplemental viral therapeutic agent for selective inhibition of tumor growth in anti-cancer therapy especially when other therapies stop working.

## Introduction

Although there have been great advances in cancer treatment over the past few decades, there still exists a number of major hurdles that limit the use of some of these new therapies. The obstacles that are presented include, but are not limited to, 1) serve side-effect due to non-specific cytotoxicity of the therapies, 2) development of drug resistance, and 3) development of tumor resistance to apoptosis. Therefore, any anti-cancer agent that could either avoid or override the shortcomings of some of the current anti-cancer treatments would be of great importance for the future design of anti-cancer therapy. Ideally, an effective anti-cancer agent should have the following properties. It should be able to 1) block only abnormal cell proliferation that results from loss of normal cell cycle regulation, e.g., cellular checkpoint mutations; 2) induce sustained cell cycle arrest that could lead to cell death or apoptosis; 3) induce specific cell killing to cancerous cells with minimal or no effect on normal cells; 4) be able to override apoptotic resistance, a typical feature of many cancerous cells; 5) have the cell killing effect independent of common oncogenic mutations such as p53; 6) confer the same arresting and cell killing effects in drug resistant cells, i.e., when other chemotherapeutic drug fails; and 7) absorbed readily or secreted by cells at its original bioactive form.

The viral protein R (Vpr) made by the human immunodeficiency virus type 1 (HIV-1) could potentially fulfill many of the aforementioned criteria. Because, 1) Vpr blocks proliferation of cancerous cells by induction of cell cycle G2 arrest [1], [2], and this arresting effect is independent of the classic DNA checkpoints [3], [4]; 2) Vpr induces apoptosis through multiple pathways that do not depend upon host cellular responses [5], [6]; and 3) Vpr selectively kills fast growing cells in a p53-independent manner [7], [8], suggesting Vpr-induced cell death may offer more selectively power in killing cancerous cells than normal cells and this cytotoxic property should be effective in many of the cancers, in which p53 gene is mutated. 4) Vpr itself is not infectious; 5) Vpr is a soluble protein that is also naturally transduced. The protein's natural tranducing ability allows it to be up taken and secreted by cells without the aid of any special delivery vehicle [9]; and 6) Vpr is a virion-associated protein. This allows possible delivery of the Vpr protein to target specific tumors such as neuroblastoma or glioma using specially designed viral vectors [For reviews, see [10]].

HIV-1 Vpr is a small accessory viral protein with an average length of 96 amino acids and a calculated molecular weight of 12.7 kD. Vpr is a unique protein that shows no known homology to any other current cellular proteins. A tertiary structure of Vpr proposed on the basis of NMR analysis consists of an α-helix-turn-α-helix domain in the amino-terminal half between amino acids 17 to 46 and a long α-helix from amino acids 53 to 78 in the carboxyl-terminal half [11]. These three α-helices are folded around a hydrophobic core in a structure that allows interaction between Vpr and other different cellular proteins [12]. The interactions between Vpr and cellular proteins underline the multifaceted roles in its interference with host cellular functions as described above [For reviews, see [8], [13], [14]].

One of the most difficult cancers to treat is neuroblastoma. This deadly sarcoma consists of malignant neuroblast cells that either appears in the autonomic nervous system or in the adrenal medulla. Neuroblastoma is considered a type of neuroepithelial tumor that mainly affects infants and children up to 10 years of age. It is one of the most common solid tumors of early childhood. The tumor typically originates in the adrenal medulla, with the most common site being the abdomen (near the adrenal gland) but it can also be found in the skin, chest, neck, pelvis, or other areas. While it is understood that genetic mutations either during fetal development or those that have been inherited lead to neuroblastoma, the exact cause of these genetic mutations are still unknown. Treatment options that are available primarily include surgery, chemotherapy, radiation therapy, and bone marrow transplants. However, despite various therapy options, the morbidity of this cancer is still very high and is currently close to 100%. The primary reason for such high morbidity is because most patients have widespread disease at diagnosis and these tumors develop rapid resistance to chemotherapy and radiation therapy.

A primary approach for cancer treatment often involves chemotherapy. Chemotherapy acts by killing rapidly divided cells, which is a main property of cancer cells. Unfortunately, neuroblastoma develops resistance rapidly to chemotherapy agents and makes treatment difficult. There are several possible reasons for chemotherapy resistance, they include 1) cells that are not killed by chemotherapy mutate and become resistant, 2) proteins that transport drugs into cancerous cells stop working, 3) cells may begin to pump drugs out of the cell as fast as they can enter the cell, 4) genes that trigger an overproduction of proteins that render a drug ineffective may become amplified, and 5) cancerous cells may begin to repair DNA breaks caused from treatment. Considering rapid drug resistance of neuroblastoma and a limited numbers of drugs currently available for the treatment of neuroblastoma, there is a great need for a new therapy that could continue eliminating malignant cells when those cells developed resistance to other anticancer drugs. A possible new solution to this dilemma is the potential use of HIV-1 Vpr with its unique biological features described above.

To demonstrate the potential utility of Vpr as an anti-cancer agent, we have carried out proof-of-concept preliminary studies *in vitro* and *in vivo*. Results of our studies showed that Vpr induces cell cycle G2 arrest and apoptosis in a large variety of cancer types. We have further demonstrated that the same Vpr effects can also be detected in cancer cells that are resistant to anti-cancer drugs such as DOX. Furthermore, we have adopted a neuroblastoma mouse model system that allows us to show the suppressive effect of Vpr on tumor growth *in vivo*.

## Results

### Vpr induces cell cycle G2 arrest in various cancerous cells

**Figure 1:**
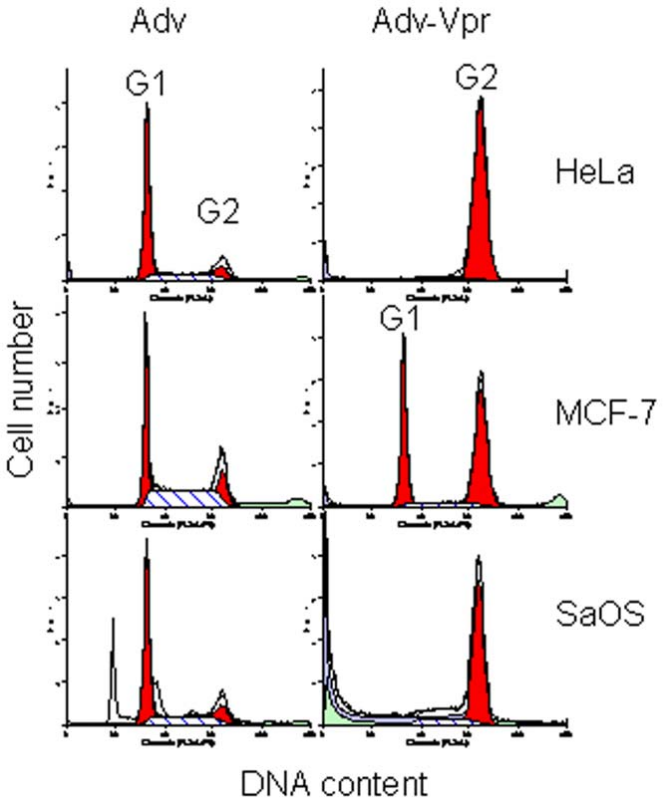
Vpr induces cell cycle G2 arrest in various cancer cell types. All cancer cell lines (see Table 1 for details; only 3 cell lines are shown here as examples) were grown as described in the Materials and Methods. Cells were transduced with Adv or Adv-Vpr with MOI of 1.0. The cells were harvested 48 hrs post-infection (*p.i.*), Cells were then prepared as described in Materials and Methods. Cellular DNA content was analyzed by FACScan flow cytometry (Becton Dickinson) as we previously described [4], [19]. The cell cycle profiles were modeled using ModFit software (Verity Software House, Inc.).

Vpr has been observed to induce cell cycle G2 arrest in a broad assortment of eukaryotic cells ranging from fission yeast to human cells [15], [16]. These observations are suggestive that highly conserved cellular activities in eukaryotic cells are indeed affected by Vpr. In this study, the adenoviral infection system was selected to measure the potential effect of HIV-1 on cell cycle G2 induction in various cancer types (Table 1) as we described previously [17]–[19]. The adenoviral systems allows for nearly 100% of cells to be infected by transduction and detection of Vpr effects are able to be observed as early as 5 hours [18]. Using this system, we tested the potential effect of Vpr on various types of cancer cell lines. These cancer types include cervical (HeLa), breast (MCF-7), ovarian (A2780), and T- or B-cell lymphomas (Jurkat and SU-DHL-4). As shown in Figure 1 and Table 1, Vpr induced cell cycle G2 arrest in all cancer cell types tested with the exception that only partial G2 shift was observed in the MCF-7 cells (Figure 1 - middle). The functional relevance of this partial G2 induction is surmised in discussion. In addition, this G2 induction appears to be independent of p53 gene status, which is consistent with a number of previous reports [7], [20].

**Table 1:** Quantitative summary of Vpr-induced G2 arrest in various cancer types.

Cell Line	Cancer Type	P53 Status	Treatment	G2 Arrest	Reference
HeLa	Cervical Cancer	Null	Adv	(−)	[44], [45]
			Adv-Vpr	(+)	
SK-N-SH (WT)	Neuroblastoma	Wt	Adv	(−)	[46]
			Adv-Vpr	(+)	
			Adv-F34IVpr**	(+)	
SK-N-SH (DOX-R)			Adv	(−)	
			Adv-Vpr	(+)	
			Adv-F34IVpr**	(+)	
MCF-7* (WT)	Breast Cancer	Wt	Adv	(−)	[33], [47]
			Adv-Vpr	(+/−)	
MCF-7 (DOX-R)			Adv	(−)	
			Adv-Vpr	(+/−)	
SaOS-2 (WT)	Osterosarcoma	Null	Adv	(−)	[48]
			Adv-Vpr	(+)	
SaOS-2 (DOX-R)			Adv	(−)	
			Adv-Vpr	(+/−)	
A2780	Ovarian Cancer	Wt	Adv	(−)	[49]
			Adv-Vpr	(+)	
SU-DHL-4	B-Cell Lymphoma	Mutant	Adv	(−)	[50], [51]
			Adv-Vpr	(+)	
Jurkat	T-Cell Lymphoma	Null	Adv	(−)	[52], [53]
			Adv-Vpr	(+)	

**Note:** *, PP2A is mutated [33], [34]; **, F34IVpr causes cell cycle G2 shift but no cycling arrest [18], [27], [28]; WT, wild type; DOX-R, doxorubicin-resistant; G2 arrest: (+), strong G2 arrest; (−), no G2 arrest, (+/−), attenuated G2.

### Vpr induces cell cycle G2 arrest and cell death regardless of drug resistance status to doxorubicin

**Figure 2:**
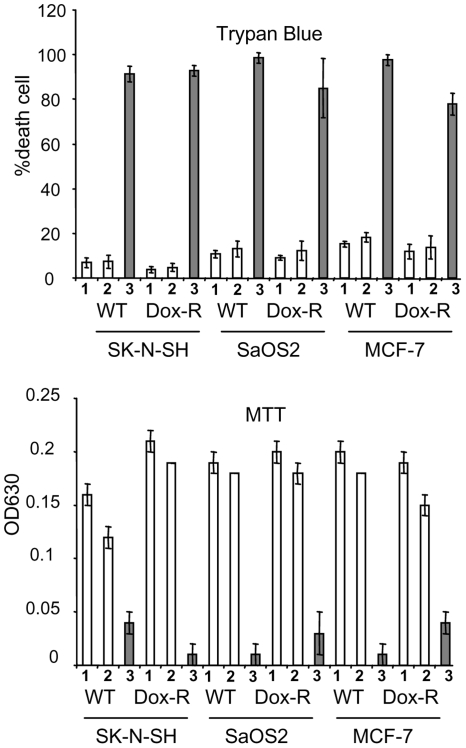
Expression of Vpr leads to cell death in a variety of wide type (WT) and doxorubicin (DOX)-resistant cancer cells as indicated. Three pairs of drug naïve (WT) and DOX-resistant (DOX-R) cancer cells were tested for Vpr-induced G2 arrest (Table 1) and cell death. These cancer cell lines were grown as described in the Materials and Methods. Cells were transduced with Adv or Adv-Vpr with MOI of 2.5. Cell viability was determined either by the Trypan Blue exclusion assay, which identifies dead cells (top figure) or by the MTT assay to measure cell survival (bottom figure). Cells were examined 5 days *p.i.* Intials: WT, wildtype; Dox-R, doxorubicin-resistant; 1, Mock; 2, Adv and 3, Adv-Vpr.

Doxorubicin (Adriamycin, hydroxydaunorubicin, DOX) is an anticancer drug that has been used in treating a wide range of cancers, including breast, stomach, lung, ovarian and bladder cancers as well as leukemia, many types of carcinoma, and soft tissue sarcomas. The precise molecular mechanism of DOX has not been well established. However, it is known to intercalate DNA and interrupt DNA replication thus leading to cell death [21]. Even though DOX continues to be an essential component of first-line anti-cancer therapies in treating many types of tumors, development of tumor drug resistant to DOX and other anticancer regimens is a major obstacle for achieving successful anti-cancer treatments [22], [23]. To explore whether Vpr can block cell proliferation by G2 arrest and kill those anti-cancer drug-resistant tumor cells, we expressed the *vpr* gene *via* Adv-Vpr transduction in three pairs of drug naïve (WT) and DOX-resistant (DOX-R) cancer cells. They are human neuroblastoma (SK-N-SH), osteosarcoma (SaOS2), and breast adenocarcinoma (MCF-7). These DOX-resistant cell lines were generated by continuous incubation of parental cell lines with stepwise increases of DOX concentrations ranging from 10^−9^ to 10^−6^ M over a period of 3 to 6 months. Detailed method for generating these DOX-resistant cell lines have been described previously [24]. As shown in Table 1, expression of Vpr causes cell growth arrest in all cells tested regardless of the drug resistant status to DOX. Moreover, measurement of cell proliferation and viability by the MTT assay, which metabolizes tetrazolium salt (MTT), showed little cell proliferation or viable cells left 5 days after Adv-Vpr transduction (Figure 2); similarly, determination of cell membrane integrity by trypan blue, which stains only dead cells, indicated that Vpr confers very potent cytotoxicity to those cells (Figure 2).

### Vpr induces dose-dependent cell death and apoptosis in DOX-naïve and resistant neuroblastoma cells

To further understand Vpr-induced cell killing in drug naïve and resistant tumor cells and to prepare for an *in vivo* study of the potential Vpr's effect on tumor growth in a mouse model, we decided to focus our study effort on neuroblastoma. Neuroblastoma was chosen as a model system because neuroblastoma is one of the most common solid tumors of early childhood usually found in babies or young children. Another reason for choosing the neuroblastoma model is that the human neuroblastoma xenograft mouse model system is well established [25], [26].

**Figure 3:**
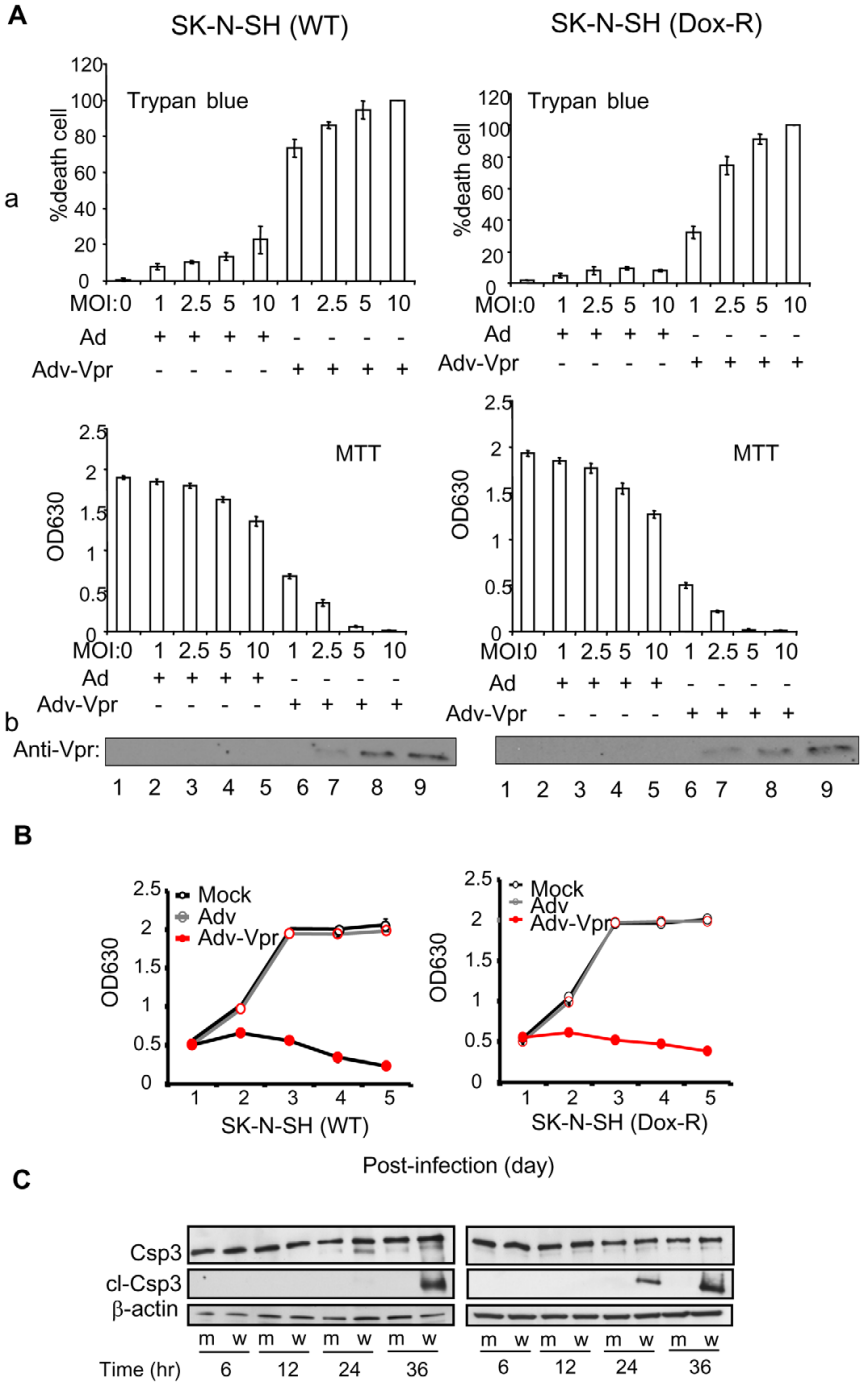
Vpr induces cell death and apoptosis in DOX-naïve and resistant SK-N-SH cells. **A.** Vpr induces dose-dependent cell death in drug-naïve and resistant SK-N-SH cells. **a.** Level of Vpr-induced cell death was examined by infecting DOX-naïve and resistant SK-N-SH cells using increasing MOI of Adv or Adv-Vpr viruses. Cell death was measured by determining the cell membrane integrity and proliferation using Trypan blue straining (left) or cell viability by the MTT assay (right). Cells proliferation and viability were examined 5 days *p.i*. **b.** Vpr protein production was confirmed by Western blot analysis. Note that it is very difficult to detect Vpr protein at low MOI. Successful infection of Adv-Vpr was verified by enlarged nuclei of cells as previously reported [43]. **B.** Vpr induces cell death over time in drug- naïve and resistant SK-N-SH cells. Both drug-naïve and resistant SK-N-SH cells treated the same way as **A**. Only MOI2.5 was used here. **C.** Vpr induces apoptosis in drug-naïve and resistant SK-N-SH cells. Caspase-3 cleavage was monitored up to 36 hours. Initials: Csp3, caspase-3; cl-Csp3, cleaved caspase-3; m, Adv-VprF34I; w, Adv-Vpr.

Before conducting *in vivo* mouse studies, we first determined the potential dose-dependent responses of Vpr-induced cell killing in both wild type (WT) and drug resistant (DOX-R) SK-N-SH neuroblastoma cells. The trypan blue and the MTT assays were used to measure cell proliferation and viability 5 days after Adv control and Adv-Vpr transductions. Summary of these results are shown in Figure 3A–a. While the increase of multiplicity of infectivity (MOI) of Adv control did not cause significant cell death, a clear dose-dependent cell killing was shown in both the WT and DOX-R cells as indicated by the trypan blue assay. At low end, MOI 1.0 caused about 40–80% cell death; whereas all of the cells (100%) were killed by MOI 10.0. The MTT assay showed the corresponding dose-dependent decrease of cell proliferation and survival of cells, and the Western blot analyses confirmed that Vpr was produced in those Adv-Vpr-infected cells (Figure 3A–b). To further understand the dynamics of Vpr's effect on cell proliferation, cell proliferation and viability was measured over time (up to 5 days) with MOI 2.5. As shown in Figure 3B, mock or Adv-infected cells showed normal cell proliferation and reached a plateau after 3 days with little or no cell proliferation detected in Adv-Vpr infected cells. The reduced metabolic activity over time suggested increased cell death over time. To further evaluate the mode of Vpr-induced cell killing in neuroblastoma cells (whether or not Vpr kills those cells by apoptosis or other mechanism) potential caspase-3 cleavage effect was measured as an indication of apoptosis. To distinguish Vpr-induced cell death from its effect on cell cycle, a Vpr mutant (F34I) that causes G2 induction but does not induce cell death was also included in this study as a control [18], [27], [28]. After adenoviral infection, status of caspase-3 was monitored starting from 6 to 36 hours by Western blot analysis using antibodies against total caspase-3 (Figure 3C, top lane) or specifically cleaved caspase-3 (Figure 3C - middle lane). No caspase-3 cleavage was detected in the Adv-F34I Vpr-infected cells (indicated by “m”) over the entire test period; the caspase-3 cleavage was clearly seen from 24–36 hours in cells that were infected with the wild type (indicated by “w”) Vpr.

Together, results of these *in vitro* studies support the idea that Vpr blocks neuroblastoma cell proliferation by cell cycle G2 arrest and cell killing *via* apoptosis. More importantly, Vpr confers these cytotoxic effects to neuroblastoma cells at a comparable level regardless of their drug resistance status.

### *In vivo* effect of Vpr on neuroblastoma tumor growth in a mouse model system

**Figure 4:**
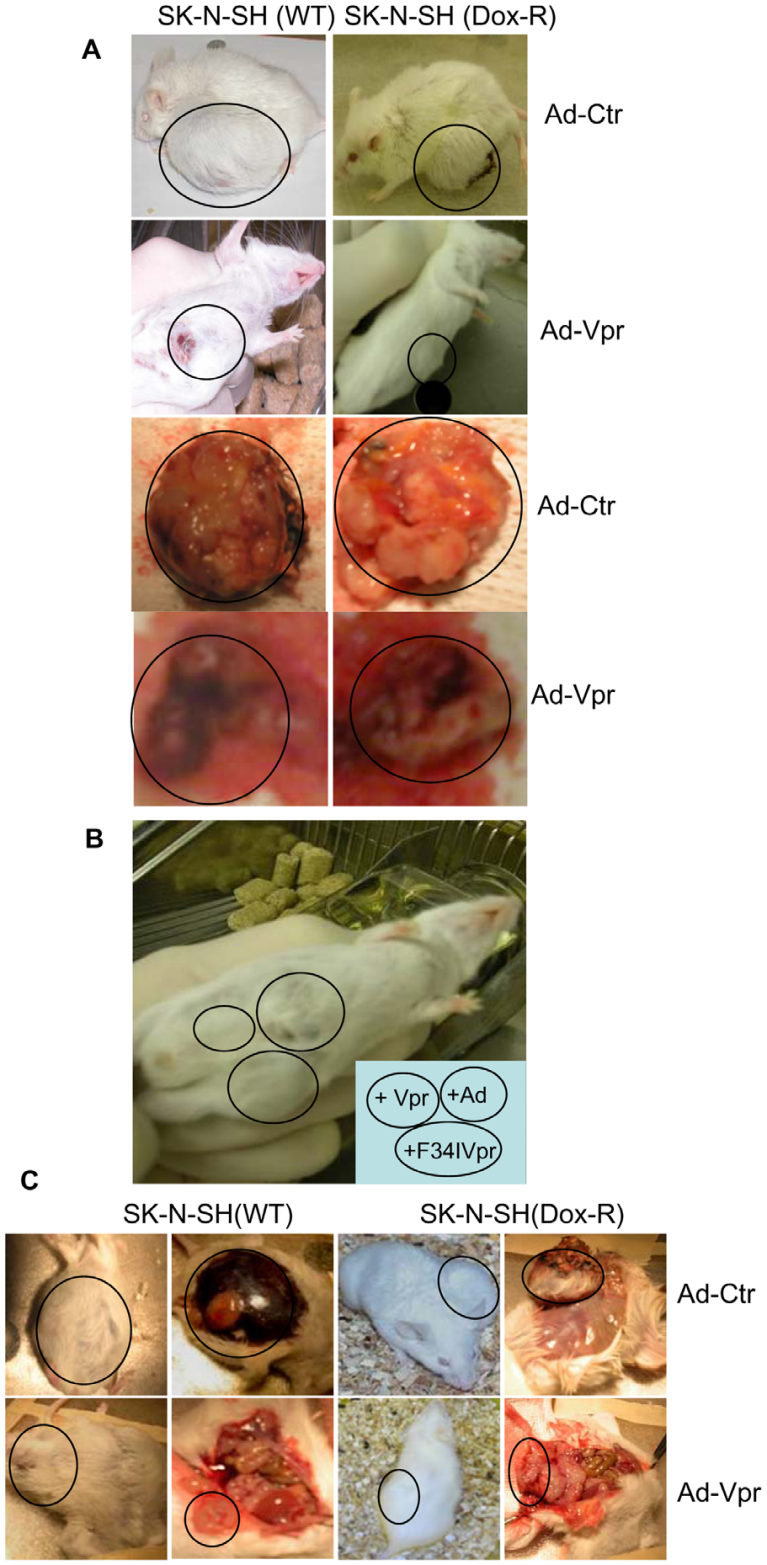
Vpr suppresses tumor regression in a neuroblastoma mouse model. Suppression of neuroblastoma tumor growth by Vpr is demonstrated here either by pre-transduction of Adv-Vpr (**A-B**) or post-intratumoral injection (**C**). For pre-transduction, wild type (WT) or DOX-resistant SK-N-SH were grown in DMEM supplemented with 10% FBS at 37°C with a 95%Air/5%CO_2_ atmosphere. Fresh cells were first grown in a 12-well plate for 36 hours and adenoviral transduction was carried out 5 hours before cell inoculation with MOI of 2.5, which was determined empirically. Cells were then treated with Trpsin- EDTA, re-suspended in DMEM and washed with PBS 3 times. Final cells were suspended in DMEM for inoculation. About 2×10^6^ cells in the volume of 100 µl were injected *s.c.* in the left flank of C57-SCID mice. 3–4 mice were injected for each treatment. These treatment groups include an Adv viral control, Adv-Vpr and Adv-F34IVpr. The F34IVpr mutant was used here as a control since a single amino acid change of amino acid 34 from Phenylalanine (F) to Isoleucine (I) renders Vpr unable to cause apoptosis (Figure 3C) but allows for the cell cycle to enter a prolonged G2 phase [18], [27], [28]. The tumor size was measured every 7 days by measuring two perpendicular tumor diameters using calipers. Final tumor measurement was at 26 days post-transduction and mice were then sacrificed for further analysis. For intra-lesional injection of Vpr, the WT and DOX-R SK-N-SH neuroblastoma cells were prepared essentially the same way as described above. 200 µl of the Adv, Adv-Vpr or Adv-F34Ivpr was then injected discretely 3-times into the tumors 2 weeks after cell inoculation with a viral concentration of 1,012/ml. The tumor size was measured every 7 days. Final measurement of tumor size was at 39 days post-injection and mice were then sacrificed for further analysis. Three independent experiments were carried out and results of these experiments with average tumor size with standard deviation (SD) are presented in Table 2.

We next examined the Vpr effect on tumor growth of neuroblastoma cells in the C57-SCID mouse model system [29], [30]. Two different approaches were used to introduce Vpr into tumors, i.e., pre- viral transduction prior to cell inoculation into mice or post-intratumoral injection. For pre-transduction, wild type and DOX-resistant SK-N-SH were injected with Adv, Adv-VprF34I and Adv-Vpr *s.c.* in the left flank of C57-SCID mice. The tumor size was measured every 7 days. Final tumor size measurement was at 26 days post-transduction and mice were then sacrificed for further analysis. As shown in Figure 4A, an average tumor size of approx. 2 cm was clearly seen in mice that were inoculated with WT or DOX-resistant SK-N-SH cells 26 days post-transduction. Similar tumor sizes were also observed in Ad-F34Ivpr infected mice. In contrast, very small nodules at the site of injection were seen in the Ad-Vpr-transduced mice, suggesting expression of Vpr in those cells prevented their growth in C57-SCID mice. To minimize potential variation of individual mice, individual mice were injected with all three transducing viruses *s.c.* at the abdominal area as shown in Figure 4B. Similar to what we have observed in mice inoculated with single treatment, about equal size of tumors were seen at the sites of Adv or Adv-F34Ivpr injections whereas little or no nodules were seen at the sites of Adv-Vpr injections.

One of the potential arguments about the results generated from pre-transduction is that expression of Vpr *in situ* could rapidly kill the SK-N-SH cells thus preventing tumor formation and thus testing of the Vpr effect on actual tumor growth. To circumvent this issue, an alternative post-intratumoral injection method was used. The WT and DOX-R SK-N-SH neuroblastoma cells were prepared and inoculated essentially the same way as described above. The Adv, Adv-Vpr or Adv-F34Ivpr was then injected discretely 3-times into the tumors 2 weeks after cell inoculation. The tumor size was measured every 7 days. Final measurement of tumor size was at 39 days post-injection and mice were then sacrificed for further analysis. Statistic tests were carried out to evaluate potential differences in tumor size each week or during the entire experimental period. The final results are presented in Table 2. Even though Vpr reduced 44.1±11.8% of the tumor growth in the wild type SK-N-SH tumors by week 1 after intratumoral injection, statistical analyses indicated a *t*-value of 2.35 (p = 0.10), i.e., no statistical difference; similarly, no tumor growth difference was seen in the Dox-R tumors at week 1. However, statistic significant differences were observed both in the wild type and Dox-R tumors by week 2 and week 3. In particular, in the WT neuroblastoma cells, Vpr reduced 64.2±6.7% or 76.4±5.9% of the tumor growth by week 2 or 3 after intratumoral injection, respectively; similarly, a comparable percent of reduction (67.1±4.5% or 75.6±1.8%) was also detected in the DOX-R cells in the same time period. Comparison of the tumor growth during the entire experimental period by using the weighted average sums [31] suggested overall differences in tumor growth between the Ad control-treated mice and Ad-Vpr injected mice (p<0.05). In contrary, no statistic differences were found between the Ad control and Ad-F34IVpr groups. Therefore, it is clear that expression of Vpr in tumors has significantly reduced the tumor growth and expression of Vpr indeed suppresses tumor growth of human neuroblastoma cells regardless of its drug resistant status.

**Table 2:** Summary of Vpr-induced tumor regression of WT and DOX-resistant neuroblastoma in C57-SCID mice.

SK-N-SH	Tumor size in mm (WT)			Tumor size in mm (DOX-R)		
	Average±SD	Size increase (%± SD)	Reduction by Vpr (%± SD)	Average±SD	Size increase (%± SD)	Reduction by Vpr (%± SD)
			**Week 0**			
Ad	983.3±15.3	n.a.	n.a.	983.3±5.8	n.a.	n.a.
**Ad-Vpr**	**973.3±15.3**	**n.a.**	**n.a.**	**986.7±5.8**	**n.a.**	**n.a.**
Ad-F34IVpr	980±26.5	n.a.	n.a.	983.3±11.6	n.a.	n.a.
			**Week 1**			
Ad	1331.3±121.3	35.8±14.4	n.a.	1217.7±24.8	23.8±2.3	n.a.
**Ad-Vpr**	**1156.7±51.3**	**18.8±4.6**	**44.1±11.8**	**1226±54.8**	**24.3±5.2**	**−1.4±15.0**
Ad-F34IVpr	1370±147.3	39.9±15.3	12.8±17.0	1220±85.4	24.0±7.4	−2.1±33.9
			**Week 2**			
Ad	2032.3±101	106.8	n.a.	2218.7±105.1	125.7±11.4	n.a.
**Ad-Vpr**	**1340±52.9**	**27.6±3.7**	64.2±6.7**	**1393.3±63.5**	**41.2±5.6**	67.1±4.5**
Ad-F34IVpr	1940±103.9	98.1±13.4	7.7±12.1	2210±95.4	119.9±5.1	4.5±6.8
			**Week 3**			
Ad	3671.3±651.4	273.6±68.0	n.a.	3560±262.9	262.1±27.6	n.a.
**Ad-Vpr**	**1576.7±6.4**	**62.0±1.9**	76.4±5.9*	**1616.7±77.7**	**63.8±7.0**	75.6±1.8**
Ad-F34IVpr	3696.7±784.2	278.8±91.4	−0.1±8.1	3630±130	269.3±17.7	−3.0±3.9

**Note:** Tumor sizes were measured at 39 days post-intratumoral injection (Week 0). Levels of statistical significance of the t-test results between the Adv control and the testing groups (Ad-Vpr or Ad-F34IVpr): *, p<0.05; **, p<0.001; Weighted average sums of the t-tests [31] for both wild type and Dox-R mice showed statistic differences at the level of p<0.05. Note: na, non-applicable.

## Discussion

In the present study, we demonstrated that a HIV-1 viral protein Vpr blocks cell proliferation by arresting those cells in G2 phase of the cell cycle in various cancer cell types tested (Table 1; Figure 1). In addition, Vpr induces cell death in three of those cancer cell lines regardless whether they are resistant or sensitive to DOX (Figure 2). Further analyses of the Vpr effect on neuroblastoma cells indicated that Vpr induces cell death in a dose-dependent manner (Figure 3A–B) *via* apoptosis as indicated by caspase-3 cleavage (Figure 3C). By using a neuroblastoma mouse model, we have further demonstrated that delivery of Vpr *via* adenoviral delivery to neuroblastoma tumors, either by pre-infection (Figure 4A–B) and intratumoral injection (Figure 4C), markedly inhibited tumor growth. Thus the described results demonstrate by proof-of-concept that Vpr might be a good candidate for further investigation on its role in tumor suppression especially in those that are resistant to anticancer drugs.

Even though Vpr induces cell cycle G2 arrest in all of the cancer cell types tested, it was noticed that the level of G2 arrested cells in the breast cancer cell line MCF-7 was much reduced (Figure 1 - middle). It is unclear at the moment why this cell line is partially resistant to Vpr-induced G2 arrest. However, there does appear to be an interesting correlation between Vpr-induced G2 arrest and the enzymatic status of protein phosphatase 2A (PP2A). As we have reported previously, one of the unique features of Vpr-induced G2 arrest is that it requires the function of PP2A [2], [4], [16], [32]. Search of literature indicated that the A regulatory subunit of PP2A is either significantly reduced or lost in the MCF-7 cell line [33], [34]. The reduction of G2 arrest in MCF-7 cells supported the notion that Vpr inhibits cell proliferation by induction of G2 arrest through a PP2A-mediated mechanism [4], [32]. Furthermore, this observation suggests that Vpr may not be able to block cell proliferation by G2 arrest in all tumor cells, especially those tumor cells that contain PP2A-related mutations. Further investigation of this notion is certainly warranted especially in the context of using Vpr as an anti-cancer agent.

Vpr induces cell death and apoptosis through multiple mechanisms [For reviews, see [5], [6]]. Since Vpr is able to induce cell death and apoptosis in both DOX-naïve and resistant neuroblastoma cells, one or few of those proposed Vpr pathways may have caused the observed cytotoxicity. Our future effort will focus on testing which mechanism of Vpr-induced cell death could overcome apoptotic resistance, a typical feature of many cancerous cells including neuroblastoma. Based on data shown in Figure 3C, it is clear that Vpr is able to cause apoptosis as indicated by the caspase-3 cleavage. This observation is consistent with our characterization of Vpr-induced apoptosis in the same neuroblastoma cells shown by using the Annex V assay [35]. There, we have further demonstrated that Vpr-induced apoptosis in the SK-N-SH neuroblastoma cells through a mitochondria-dependent and caspase-9-mediated mechanism [35].

We used pre-infection and intra-tumoral injections in our mouse model for the introduction of Vpr into neuroblastoma tumors. These two methods were used because expression of Vpr *in situ* could rapidly kill cells and thus prevent testing on actual tumor growth. Our results demonstrated both methods significantly reduced tumor growth after introduction of Vpr. Comparably, DOX-resistant cells also demonstrated similar level of tumor size reduction supporting the premise that Vpr expression suppresses tumor growth regardless of drug resistant status.

It is worthwhile to mentioning that, in the described experiments, we did not test the potential Vpr effect on metastatic neuroblastoma cells. We did, however, carry out preliminary pharmacological studies to test potential adverse effect of C57-SCID mice when they were exposed to Vpr through systematic full-body injection. In those experiments, an escalating increase of Adv-Vpr viral particles ranging from 1×10^13^ to 6×10^13^ of viral particles were injected into C57-SCID mice through tail veins. No obvious adverse reactions were observed in those mice during the entire experimental period. Pathologic examinations of various organs 3 weeks after viral injection showed no clear cytotoxic effects imposed by Vpr (data not shown). Therefore, systematic introduction of Vpr could also be an option for future testing.

The concept of using HIV-1 Vpr as a potential anticancer agent is not new. In fact, other early studies including ours have suggested this possibility [36] and a number of studies have showed that delivery of Vpr to various tumors such as melanoma [37]–[39], hepatoma [40], [41] and oral cancer [42] suppress tumor growth *in vivo*. What is new, however, is that we have demonstrated for the first time that Vpr is capable of suppressing tumor growth regardless whether it is sensitive or resistant to an anticancer drug through induction of cell cycle G2 arrest and apoptosis. This finding could potentially be significant because destruction of drug resistant cancer cells makes it a potential and powerful new and alternative anti-cancer agent. This type of anticancer agent may become particularly useful as a supplemental viral therapeutic agent for selective inhibition of tumor growth especially when other therapies fail.

## Materials and Methods

### Cell lines and culture

Various human cancer cell lines that represent different types of cancers with or without drug resistance to the anticancer drug doxorubicin (DOX) are used in this study and summarized in Table 1. Unless specifically specified, these cell lines were grown in the Dulbecco's Modified Eagle's Medium (DMEM) (Cellgro) or RPMI 1640 medium (Sigma) supplemented with 10% fetal bovine serum (FBS, Invitrogen). Incubation was at 37°C in a 5% CO_2_ incubator.

### Adenoviral infection

Adenoviral vector (Adv) and Adenoviral vector inserted with the *vpr* gene (Adv-Vpr) were kindly provided by Dr L. J. Zhao [17]. Approximately 1×10^6^ of the test cells were infected with Adv or Adv-Vpr viruses. Forty-eight hours *p.i.*, cells were harvested for cell cycle analysis or Western blot analysis. Infections were performed at a multiplicity of infection (MOI) of 1.0 for cell cycle analyses as shown in Table 1 and Fig. 1. MOI of 2.5 was used for initial cell death analyses as shown in Fig. 2.Under this adenoviral transducing condition, greater than 90% infection efficiency was achieved with these viral stocks (data not shown).

### Cell cycle analysis

The cells were harvested 48 hrs *p.i.*, then washed twice using 2 ml 5 mM EDTA/PBS and centrifuged at 1,500 rpm. Following centrifugation, cells were re-suspended in 1 ml 5 mM EDTA/PBS and fixed with 2.5 ml of 95–100% cold ethanol. Fixed cells were then stored overnight at 4°C. Following storage, cells were again centrifuged and washed twice with 2 ml 5 mM EDTA/PBS and centrifuged again at 1,500 rpm. After re-suspension in 0.5 ml PBS, cells were incubated with RNase A (10 µg/ml) at 37°C for 30 minutes and then at 0°C with propidium iodine (PI, 10 µg/ml) for 1 hour. Cells were then filtered and DNA content was analyzed by FACScan flow cytometry (Becton Dickinson). The cell cycle profiles were modeled using ModFit software (Verity Software House, Inc.).

### Western blot analysis

Cells were lysed with lysis buffer (50 mM Tris, pH 7.5, 150 mM NaCl, 2 mM EDTA, 1% Triton X-100) for 30 minutes while on ice. Cells were then centrifuged at 13,000 rpm for 1 minute to remove debris. Protein concentrations recovered in the supernatants were measured using BCA protein assay kit (Pierce). After boiling, 50 µg of protein was loaded into Criterion Precast Gels (BioRad) for electrophoresis separation. Proteins were then transferred to Trans-blot® Nitrocellulose membranes and blocked with 5% skim milk in TBST buffer (10 mM Tris, pH 8.0, 150 mM NaCl, 0.1% Tween 20) for 1 hour at room temperature. Primary antibodies were applied overnight at 4°C. After washing 3 times in TBST for 10 minutes each time, the membranes were then incubated with secondary antibody for 1 hour at room temperature. Antibodies used included rabbit polyclonal anti-Vpr [custom generated through the Proteintech Group, Inc (Chicago, IL)], rabbit polyclonal anti-caspase 3 (Cell Signaling and rabbit polyclonal anti-cleaved-caspase 3 (Asp175) (Cell Signaling) antibodies. After incubation with secondary antibody, membranes were washed again and proteins were detected with Supersignal® West Dura Extended Duration Substrate (Pierce, Rockford, IL).

### MTT assay

Cell viability was determined by the MTT assay (Boehringer Mannheim) following the manufacturer's instructions. Approximately 1×10^6^ of the testing cancer cells were used to initiate the experiments. On day one, cells were trypsinized and centrifuged at 500 rpm for 5 minutes. The media was then removed and cells were re-suspended in 1 ml of complete media and a cell count per ml was calculated. Using this cell count, cells were then diluted to 75,000 cells per ml with complete media. 100 µl of cells (7,500 cells total) were then added to each well of a 96-well plate and incubated overnight at 37°C. On day two, cells were treated with Adv or Adv-Vpr transduction. At the day of indicated test, 20 µl of 5 mg/ml MTT was added to each well and incubated at 37°C for 3.5 hours. Next the media was removed, washed with PBS and then 150 µl of MTT solvent was added to each well. The plates were then gently agitated for 15 minutes in an orbital shaker. After agitation, absorbance at 630 nm was measured.

### Trypan blue staining

Viability of treated cells was analyzed using trypan blue stain, a vital dye (Gibco BRL). Cells were trypsinized and centrifuged at 500 rpm for 5 minutes. The media was then removed and cells were re-suspended in complete media. Next, cells were diluted to an approximate cell concentration of 1×10^5^ to 2×10^5^ cells per ml in a screw cap test tube. 0.1 ml of 0.4% trypan blue stain was then added and the test tubes were mixed thoroughly. The cell mixture was then allowed to stand for 5 minutes at room temperature. After 5 minutes cell counting was performed using a hemacytometer. Viable cells (no staining) and non-viable cells (stained blue) were observed and counted under microscope.

### Neuroblastoma mouse model

The human neuroblastoma xenograft mouse model system is well established [25], [26]. A reproducible mouse model for human neuroblastoma can be achieved typically by subcutaneous (*s.c.*) or intraperitoneal (*i.p.*) injection of 2–5×10^6^ of human neuroblastoma SK-N-SH cells into C57BL/6N (C57BL) and C57BL/6J-scid/scid (C57-SCID) (Charles Rivers). Treatment groups include mice injected with the adenoviral vector (Adv) control, wild type Vpr-carrying adenovirus (Adv-Vpr) and F34I mutant Vpr-carrying adenovirus (Adv-F34IVpr). Three mice were used in each treatment group with a total of 9 mice in each experiment. Three independent experiments were carried out for this study. Palpable tumors at the injection sites developed within 3 weeks with an average size of 2–3 cm in diameters, and tumors later metastasize into various organs including adrenal glands, local lymph nodes, bone, liver, skin, and bone marrow [29], [30]. Histologically, the tumors developed in mouse resembles the original metastases from which the tumors were derived and the SK-N-SH tumor cells can be distinguished from the *in vitro* cultured cells by the dopamine-β-hydroxylase (DBH) activity [30]. The animal protocol used in this study was approved by the Animal Care and Use Committee of the Children's Memorial Research Center (no. 2006-29).

### Statistical Analyses

The statistic *t*-test was used in log scale to analyze the potential differences in tumor sizes for each week's measurement between the Ad control group and the Vpr-treated groups (Ad-Vpr or Ad-F34IVpr). To evaluate the overall impact of Vpr on tumor growth during the entire 3 weeks, the weighted average sums of the tumor sizes (in log scales) were compared using a related test designed specifically for xenograft tumor models [31].

## Acknowledgments

The authors would like to dedicate this paper to the late Dr. Bernard L. Mirkin for his life-time dedication to study cancer biology especially in the area of drug resistance of neuroblastoma. His selfless support and guidance has been truly invaluable. The authors are also indebted to Dr. Abdelhadi Rebbaa for the described DOX-resistant cell lines, and Dr. Hongbin Fang and Ms. Ling Cai for assistance of statistical analyses.
